# 

**Published:** 1839-04-01

**Authors:** 


### BIBLIOGRAPHICAL RECORD.

1. A Letter to Dr. Chambers, F.R.S. &c. on several important Points relating to the Nature and proper Treatment of Gout. By Sir Charles Scudamore, M.D. Octavo, pp. 60. Longman and Co. Dec. 1838.

2. On Granular Degeneration of the Kidneys, and its connexion with Dropsy, Inflammation, and other Diseases. By Robert Christison, M.D. President of the Royal College of Physicians, &c. Ed. Octavo, pp. 288. Edinb. Adam & Charles Black. Dec. 1838.

*** *In our next.*

3. Medical Portrait Gallery, Part 11. Price 3s. By Mr. Pettigrew. Contains Conclusion of Memoirs of Dr. Clutterbuck—Portrait and Memoir of Huxham—Portrait and Memoir of Mr. Wardrop.

*** *This is a very good number.*

4 Medical and Physical Researches ; or, Original Memoirs in Medicine, Surgery, Physiology, Geology, &c. &c. Illustrated with numerous Plates. By R. Harlan, M.D. F.L.S. London, Surgeon to the Philadelphia Almshouse. Octavo, pp. 653. Plates. Philadelphia, 1835.

5. Elements of the Pathology of the Human Mind. By Thomas Mayo, M.D. Octavo, pp. 182. Murray, Dec. 1838.

6. Essays on the most important Diseases of Women. By Robert Ferguson, M.D. Professor of Obstetric Medicine, &c. King's College, London. Part I. Puerperal Fever. Octavo, pp. 300. Murray, Dec. 1838.

7. An Exposition of Quackery and Imposture in Medicine; being a Popular Treatise on Medical Philosophy. By Dr. Ticknor. With Notes by W. Wright, Surgeon-Dentist. James S. Hodson, 112, Fleet Street. 1838.

8. A general Outline of the Animal Kingdom. By Thomas Rymer Jones, F.Z.S. Professor of Comparative Anatomy at King's College. Part III. Price 2s. 6d. Illustrated by numerous Engravings. Jan. 1839. J. Van Voorst, Paternoster Row.

9. Elements of Physiology. By J. Muller, M.D. Translated from the German, with Notes by W. Baly, M.D. Illustrated with Steel Plates, &c. Part IV.—containing Ciliary Motion, Mucular and Allied Motion, Voice and Speech. Taylor and Walton. Price 4s. Jan. 1839.

10. Stammering, practically considered: with the Treatment in detail. By T. Bartlett, Assistant-Surgeon in the Army. Small octavo, pp. 83. Sherwood and Co. 1839.

11. An Improvement in the Pathology and Treatment of Small-pox. By Robert Stevens, M.R.C.S. 1838.

12. Illustrations of Osteology. By Theodore S. G. Boisragon, M.D. Cheltenham. Folio. Highley, London, 1839.

13. The Dublin Medical Press—a Weekly Medical Journal, each number containing sixteen pages. Quarto, price Sixpence, stamped.

14. Statistical Report of the Richmoud Lunatic Asylum. By J. Mollan, M.D.

15. The Quarantine Laws, their Abuses and Inconsistencies. A Letter to Sir J.C. Hobhouse, Bart. By A. T. Holroyd, Esq. Simkin and Marshall, 1839.

16. The Medical Examiner. A Medical Journal, published every Fortnight, in Philadelphia. No. 11 to No. 23. May 23d to November 7th.

*** *In Exchange.*

17. The Naturalist's Library. Vol. VIII. Mammalia. Conducted by Sir W. Jardine. Bart. Lizars, Edinb. 1830.

18. Illustrations of Cutaneous Disease—a Series of Delineations of the Affections of the Skin, in their more interesting and frequent Forms; with a Practical Summary of their Symptoms, Diagnosis, and Treatment. By Robert Willis, M.D. &c. Fasciculi I. II. III. Folio. Bailliere, 1839.

19. The 25th Report of the Director of the West Riding of York Pauper Lunatic Asylum. Wakefield, 1839.

20. The Cyclopædia of Anatomy and Physiology. Part XVI. containing "Heat," by Dr. Edwards—Hermaphrodism, by Dr. Simpson—Hernia, by W. H. Porter, Esq. —Hibernation, by Dr. M. Hall.

21. Observations on the Employment of solid Nitrate of Silver in Stricture, &c. By T. B. CURLING, Esq.

22. Observations on Hypertrophy and other Affections of the Os Uteri. By EVORY KENNEDY, M.D. Master of the Dublin Lying-in Hospital.

☞ *In our next.*

23. History of British Reptiles. By THOS. BELL, F.R.S. Part II. Price 2s. 6d. J. Van Voorst.

24. A general Outline of the Animal Kingdom. By THOMAS RYMER JONES, F Z.S. Illustrated by numerous Engravings on Wood. Part IV. Price 2s. 6d. Van Voorst.

25. Portrait of Dr. J. Elliotson, delineated and lithographed by Miss Newell.

*** *A very striking likeness.*

26. Cataract, &c. By JOHN STEVENSON. Fourth Edition, improved. Highley, 1838.

27. Deafness, its Causes, Prevention, and Cure, &c. By JOHN STEVENSON, Esq. Fourth Edition. Highley, 1838.

28. The Physiology or Mechanism of Blushing; illustrative of the Influence of Mental Emotion on the Capillary Circulation; with a general View of the Sympathies, and the Organic Relations of those Structures with which they seem to be connected. By THOMAS H. BURGESS, M.D. Octavo, pp. 202. Churchill, 1839.

*** *In our next.*

29. Lectures on the Principles and Practice of Midwifery. By JAMES BLUNDELL, M.D. Edited by CHARLES SEVERN, M.D. Registrar of the Medical Society of London. One closely printed octavo volume, pp. 568. I. Masters, Aldersgate Street. 1839.

*** *We have only time, at this late period of the quarter, to quote the following sentence from Dr. Severn's preface, and in which we cordially agree.*

"*The fame of Dr. Blundell is firmly established—his successful efforts to enlarge the sphere of knowledge must ensure to these Lectures universal circulation, wherever genius is appreciated, science regarded, and humanity valued.*"

30. A Series of Anatomical Plates, &c. By JONES QUAIN, M.D. Fasciculi 58 and 59. Division 3. Nerves 20. Price 2s. each. Taylor and Walton. March, 1839.

31. Prostitution in London, with a Comparative View of that of Paris and New York, as illustrative of the Capitals and large Towns of all Countries; and proving Moral Depravation to be the most fertile Source of Crime, and of personal and social Misery. By MICHAEL RYAN, M.D. &c. &c. Small octavo, pp. 447. London, March 1839.

*** *This work came too late for notice in this number of the Journal.*

32. A Manual for Students who are preparing for Examination at Apothecaries' Hall, or other Medical Institutions. By WILLIAM MEADE, Member of the Royal College of Surgeons, London. 12mo. pp. 709. London: Renshaw, 1839.

☞ *This is a decided hit for a numerous class. Mr. Mead, we understand, is one of the most lucid popular teachers in this metropolis.*

### INDEX.

A.

Abdominal tumors, Dr. Bright on.... 194

Abdominal tumor ................ 332

Abernethiana .................... 526

Abernethy as a lecturer............ 527

Abscess, chronic, in the left iliac fossa 265

Acrid diuretics .................... 243

Action of diuretic medicines......... 243

Action of the stapedius ............ 425

Acute gastritis .................... 318

Addenbrooke's hospital, clinical contributions from ................ 299

Advantages of royalty in death-bed scenes ......................... 528

Ætiology of club feet.............. 227

Algiers, state of the faculty in ...... 534

Alterations of the substance of spleen 194

America, temperance societies in.... 166

American medical press .......... 624

Amputation for traumatic grangrene 552

Amputations, mortality after ...... 556

Anatomical drawings, by Mr. Hawkins 146

Anatomical plates, by Quain........ 174

Anatomy, recent works on ........ 173

Anatomy, descriptive and pathological, by Meckel ..................... 173

Anatomy, practical and surgical, Mr. Wilson on ...................... 174

Anatomy, Dr. Hunter on .......... 174

Anatomy of the perinæum, by Morton 174

Anatomy & physiology, Cyclopædia of 414

Anazoturia ...................... 10

Anchylosis of cervical vertebræ .... 296

Aneurysm, ventral................ 84

Aneurysm of the ventricles ........ 102

Aneurysm of the auricles.......... 109

Aneurysm of the valves of the heart 110

Aneurysm of the thoracic aorta. By Sir D. Dickson ................ 631

Aneurysms of the heart, Mr. Thurnam 102

Angular curvature of the spine...... 481

Animal and earthy matter in human bones ....................... 91

Animal kingdom, Jones on the...... 176

Animal kingdom, Jones' outline of the 522

Animal magnetism................ 630

Anomalous aneurysm of the aorta .. 545

Antigua ........................ 56

Antiphlogistic remedy, efficacy of mercury as an ................... 245

Anuria.......................... 14

Aorta, origin of, from right ventricle 591

Application of raw cotton to erysipelatous surfaces .............. 260

Armies, medical statistics of........ 411

Arms and neck, pulsations in the superficial veins of the .......... 237

Arsenic, poisoning by ............ 251

Arsenic, successful treatment of poisoning by .................... 612

Arteries, torn, why do they not bleed? 573

Ataxic form of puerperal fever...... 475

Atrophy of the liver .............. 320

Auricles, aneurysms of the ........ 109

Azoturia........................ 11

B.

Baillie's generosity... ............ 528
Ballingall on military surgery ...... 408
Barbadoes ...................... 54
Barber-surgeons.................. 165
Bartlett on stammering............ 507
Beaumont on polypus of the uterus .. 167
Beauty of form, Dr. Joslyn on the .. 143
Beemner's cases .................. 645
Bell's history of British reptiles .... 521
Bengal medical memorial . ........ 651
Berehaven, epidemic cholera of...... 289
Bigelow on pneumo-thorax ........ 552
Biographical memoir and portrait of Dr. James Johnson.............. 637
Bladder, stone crushed in, 270 years ago ......................... 629
Blood, Dr. Maitland on physiology of 69
Blood, mechanical constitution of the 70
Blood, chemical constitution of the.. 71
Blood, organic relations of the ...... 72
Blood, M. Magendie on the ........ 203
Blood, on the presence of urea in the 241
Blood, pus in the ................ 273
Blood, urea in the, in cholera ...... 536
Boisragon's illustrations of osteology 520
Bombay medical transactions ...... 73
Bond's clinical contributions........ 299
Bowring on the plague & on quarantine 163
Bricheteau's clinique ............. 598
Bricheteau on typhus fever ........ 601
Bright on abdominal tumors........ 194
British Guiana .................. 53
British birds, Yarrel's history of.. 176 521

British reptiles, Bell's history of .... 521
Brodie on medical study .......... 211
Budd on concentric hypertrophy.... 98
Burns' principles of surgery ........ 168
Bursæ, diseases of the ............ 170

C.

Calculous diseases, Mr. Hutchinson on 77
Cantharides a contra-stimulant...... 603
Carbonate of soda, remedial value of 288
Carcinoma of the pylorus...... ... 320
Cardiac disease, case ............... 74
Catarrh, chlorine inhalation in...... 231
Caustic issues in phthisis pulmonalis, 599
Cerebro-spinal phenomena, Dr. Waugh on the ........................ 174
Cervical vertebræ, anchylosis of .... 296
Chemical constitution of the blood . 71
Chemical examination of the liquor amnii ........................ 188
Chemistry, Turner's elements of .... 524
Chemistry, organic, by Profes. Liebig 524
Chlorine inhalation in catarrh ...... 231
Cholera, urea in the blood in........ 536
Chomel on pneumo-thorax ........ 232
Christison, Dr. on granular degeneration of the kidnies ............ 350
Chronic diseases, Clendinning on the vital organs in.................. 93
Chronic abscess ................. 125
Chronic hypertrophy of the scalp.... 170
Chronic scrofulous ophthalmia...... 246
Chronic abscess in the left iliac fossa 265
Claims of Hippocrates to admiration 628
Clendinning on the vital organs in chronic diseases ................ 93
Clinical contributions from Addenbrooke's Hospital .............. 299
Clinical observations on surgery .... 550
Clinical reports from the Pennsylvania Hospital ...................... 554
Clinical report, Mr. Syme's ........ 572
Clinique of M. Bricheteau ....... 598
Club feet, ætiology of ............ 227
Cochlea, functions of the ......... 419
Cock on congenital deafness ........ 177
Cogswell on the properties of iodine 116
Comparative anatomy of the nervous system, by M. Leuret............ 532
Compound fracture of the cranium.. 555
Concentric hypertrophy, Dr. Budd on 99
Concours for the chair of organic chemistry at Paris .............. 594
Congenital deafness, Mr. Cock on.... 177
Congestion, as contra-distinguished from inflammation.............. 135
Consequences of inflammation...... 123
Constitutional causes of inflammation 132
Consumptive disorders, Dr. Furnival on 157
Contagiousness of fevers .......... 225
Contraction of the lower extremities 567
Cooper on the muscles of the eye.... 333
Corpus spongiosum urethræ, fistula of 185
Corrigan on pulmonary diseases .... 316
Cowan's vital statistics of Glasgow.. [illegible]
Crampton's history of medicine .... 626
Cranium, compound fracture of the.. 555
Croupy inflammation of the mouth.. 595
Crust for the critics .............. 341
Curvatures of the spine, Mr. Hare on 478
Cusack's mode of applying the nitrate of silver to enlarged amygdalæ.... [illegible]
Cutaneous disease, illustrations of .. 48
Cyanosis ........ ............... 279
Cyclopædia of anatomy and physiolog[illegible] [illegible]

D.

Danger of being buried alive........ [illegible]

Dangers of lithotrity, Mr. Fergusson on 223

Deafness, Mr. Cock on ............ 177

Death of Vesalius ................ 526

Delirium tremens, history and treatment of ...................... [illegible]

Delirium tremens ................ [illegible]

Dendy on diseases of the skin ...... [illegible]

Derangement of the sensibility of th[illegible] stomach .................... [illegible]

Devergie on sudden death. ........ 266

Diabetes insipidus ................ 11

Diabetic blood, Dr. Rees on ........ 190

Diaphragm, rupture of the ..... .. 193

Dickson, Sir David's, case of aneurysm of the thoracic aorta ............. 631

Dieffenbach on the orthopædic establishments in Paris .............. 592

Diffuse inflammation of the scalp.... 261

Digestive functions, impairment of the 300

Diphthérite, treatment of............ 595

Discovery of the vital principle...... 443

Disease, Mr. Harrison on the philosophy of ................... .... 510

Diseases of the lungs.............. 59

Diseases of the stomach and bowels.. 60

Diseases of the skin, Mr. Dendy on .. 152

Diseases of the skin, arrangement of.. 154

Diseases of the stomach and intestines 29[illegible]

Diseases of the liver .............. 332

Diseases of the ear................ 428

Diseases of the heart, M. Gendrin on 589

Dislocation of the humerus ........ 554

Dislocation of the head of the radius backwards .................. 555

Dislocation of the femur on dorsum ilii 572

Disorders of the intestines ....... 323

Dissecting aneurysm, Dr. Goddard on [illegible]

Diuretic medicines, action of...... [illegible]

Division of labour among the Egypt[illegible]s [illegible]

Dominica ................ [illegible]

Dorsum ilii, dislocation of the femur on [illegible]

Dropsy, post mortem examination of a case of.................... 646

Druitt's surgeon's vade mecum...... 522

[illegible] on uterine hæmorrhage...... 611

[illegible]ee lunatic asylum ............ 575

[illegible]ytren's pommade against the [illegible]ling off of the hair............ 610

Dura mater, Mr. Porter on puncturing the, after trephining ............ 578

E.

Ear, Mr. Pilcher on the ............ 414

Ear, functions of the ... .......... 414

Ear, diseases of the............... 428

East Indies, treatment of hydrocele in [illegible]e ..................... 608

[illegible]ct on the pulse by change of posture 182

[illegible]acy of mercury as an antiphlogis-[illegible]c remedy ..................... 245

[illegible], pellicle of an, an application to [illegible]wounds ..................... 261

[illegible]ptians, division of labour among the ........................ 626

El[illegible]ments of physiology by Muller.... 517

Elements of chemistry, Dr. Turner's 524

[illegible]hysema, pulmonary, Dr. Lom-[illegible]rd on ..................... 397

Emphysema, vesicular .......... 398

E[illegible]physema, lobular .............. 398

E[illegible]hysema, lobar ................ 399

En[illegible]sted tumors................... 262

England, treatment of insanity in.... 278

Enlargement of the liver .......... 333

Enormous ventral aneurysm........ 84

Enteritis and colitis................ 330

Epidemic fever, Dr. Yates on........ 162

Epidemic cholera of Berehaven...... 289

Epilepsy ......................... 595

Equinia ......................... 616

Erysipelatous surfaces, application of raw cotton to ................. 260

Esquirol on mental disorders........ 33

Esquirol on insanity .............. 482

Expulsion of a long loop of the intestinal canal .................... 237

Extirpation of the parotid gland .... 550

Extraordinary operations for hæmorrhoids ........................ 574

Eye, Mr. Cooper on the muscles of the 201

Eyelids, paralysis of the. ..... .... 613

F.

Farr's medical annual ............ 164
Femur, Mr. Gulliver on shortening of the neck of the ........... .... 543
Femur, dislocation of the, on the dorsum ilii .................. .... 572
Fenner on pressure to the uterus.... 338
Fergusson on the dangers of lithotrity 223
Fergusson on puerperal fever ...... 461
F[illegible]usson on pauperism and poor-laws 504
F[illegible], statistics of, in Glasgow... .. 511
Fevers, contagiousness of.......... 225
Fistula of corpus spongiosum urethra 255
Fistula in ano..................... 574
Form, Dr. Joslyn on the beauty of .. 143
Formation of pulmonary emphysema, 399
Forcke on veratria................ 229
Foundling and bastard children ..... 249
Fracture, fortunate treatment of .... 573
Fractures, Smee on moulding tablets for 536
Functional derangement of the kidneys 6
Functional derangement of stomach, 299
Functions of the ear ............... 414
Functions of the cochlea .......... 419
Fungus hæmatodes ............... 258
Furnival on consumptive disorders .. 157

G.

Gastralgia ...................... 67
Gastric affections, predisposing causes 305
Gastric affections, treatment of...... 313
Gastro-enteric irritation .......... 463
Gastrotomy...................... 264
Gendrin on diseases of the heart .... 589
German physicians................ 630
Glanders in the human subject...... 613
Glasgow, Cowan's vital statistics of . 511
Gleanings in the Indian seas, Mr. Marshall's ......................... 633
Goddard on dissecting aneurysm .... 545
Gonorrhœal rheumatism and ophthalmia, Mr. Lawrence on .......... 549
Gonorrhœal ophthalmia .......... 549
Gonorrhœal rheumatism .......... 550
Gorham on intus-susception in infants 185
Gout, Sir C. Scudamore on ........ 530
Gout, treatment of................ 531
Gout and sinapisms .............. 540
Granular degeneration of the kidnies, Dr. Christison on .............. 350
Granville, Dr. and Dr. Roe ........ 287
Graves on various diseases ......... 539
Gray on prolapsus uteri............ 346
Grenada ........................ 54
Guiocomini on cantharides ........ 603
Gulliver on necrosis .............. 86
Gulliver on suppuration ........... 273
Gulliver on shortening of the neck of the femur .................... 543
Guy's hospital reports ............. 177
Guy on the effect upon the pulse by change of posture .............. 183

H.

Hæmatemesis..................... 321
Hæmatology ..................... 203
Hæmatophobia ................... 280
Hæmorrhoids .................... 331
Hæmorrhoids, extraordinary operations for ....................... 574
Hadwen's case of popliteal aneurysm, 80
Hallucinations ................... 496
Hare on curvatures of the spine ... 478
Harrison on the philosophy of disease, 510
Hawkins' anatomical drawings...... 146

Hearing, Dr. Todd on .... ........ 415
Heart, Mr. Thurnam on aneurysms of 107
Heart, dissecting aneurysm of the.... 107
Heart, aneurysm of the valves of the, 110
Heart, M. Gendrin on diseases of the, 589
Heart, opening between the ventricles of the .. ...................... 591
Hematosine......................... 71
Hernia, Mr. King on ............... 191
Hippocrates, claims of, to admiration, 628
History of a female having 4 mammæ 78
History and treatment of delirium tremens ........................ 269
History of British birds, by Yarrell.. 521
History of British reptiles, by Bell .. 521
History of medicine, Sir P. Crampton's 626
Holroyd on the quarantine laws .... 525
Hospital medical staffs ............ 164
Horse-hair gloves ................. 631
Human subject, glanders in the...... 613
Humerus, dislocation of the........ 554
Hunter's human anatomy.......... 174
Hunter's idea of life ...... ...... 444
Hunter's death-bed .............. 528
Hutchinson on calculous diseases.... 77
Hydatidic goitre, case of ........... 256
Hydrocele, iodine injections in ...... 605
Hydrocele, treatment of, in East Indies 608
Hydruria....... ................. 6
Hypertrophy, concentric, Dr. Budd on 98
Hypertrophy of liver.............. 335

I.

Icterus ........................... 332
Ileus, case of, in which gastrotomy was performed .................. 264
Illusions of the insane ............ 499
Illustrations of cutaneous disease, by Dr. Willis ... ................. 519
Illustrations of osteology ........... 520
Improvement of medicine, Dr. Thomson on ......... .............. 160
Incontinence of urine, treatment of with nux vomica.................. 230
Indian seas, gleanings in the........ 633
Infants, Mr. Gorham on intus-susception in........................ 185
Inflammation, Dr. Macartney on .... 119
Inflammation, history of .......... 120
Inflammation, consequences of. .... 123
Inflammation, phenomena of........ 123
Inflammation, reputed consequences of 126
Inflammation, constitutional causes of 132
Inflammation, proximate cause of . .. 135
Inflammation, on congestion as contradistinguished from .............. 135
Inflammation, remedies for . ... .. 136
Inflammation, lancet punctures in .. 169
Inflammation of the umbilical vein in infants .......................... 247
Inflammation, diffuse, of the scalp .. 261
Inflammation of the liver .......... 333
Inflammatory intus-susception...... 185
Inflammatory dyspepsia.. .......... 316
Inhalation of iodine in pulmonary disease ........................ 535
Injuries, Stevens on the primary treatment of ....................... 216
Insane, illusions of the ............ 499
Insanity, M. Esquirol on .......... 37
Insanity, causes of death in ........ 42
Insanity, dependent on cerebral alterations? ........................ 43
Insanity, treatment of ............ 48
Insanity in England .............. 279
Insanity, Ray's medical jurisprudence of........................... 431
Insanity, M. Esquirol on .......... 482
Insanity, connexion of suicide with .. 489
Insanity, treatment of ............. 491
Intellectual mania, legal consequences, 441
Intestinal sloughing .............. 75
Intestinal canal, expulsion of a long loop of the ......... .......... 251
Intestinal entozoa ................. 327
Intestines, perforation of the, by worms 233
Intestines, disorders of the ......... 323
Introsusceptio, cured by forcing air into the intestine ............... 645
Intus-susception in infants, Mr. Gorham on ....................... 185
Iodide of potassium, Mr. Laycock on the 533
Iodine, Dr. Cogswell on properties of, 116
Iodine injections in hydrocele ....... 605
Iron, utility of, in syphilitic affections, 620
Irregularities of the subclavian artery and vein ...................... 592
Ischuria renalis .................. 14

J.

Jamaica ........................... 56

Jardine's naturalist's library ........ 522

Johnstone, Dr. on the salts of silver in secondary syphilis............ 538

Johnson, Dr. J. memoir and portrait of 637

Jones' outline of the animal kingdom, 176

Jones' outline of the animal kingdom, 522

Joslyn on the beauty of form ...... 143

K.

Kidneys, functional derangement of the 6

Kidneys, granular degeneration of the, Dr. Christison on .............. 360

King on hernia ................... 191

L.

Lancet punctures in inflammation ... 169

Larynx, neuralgia of the .......... 539

Late Mr. Lockley .... ........... 284

Law of lunacy .................... 277

Law of lunacy..................... 278

Law on mercury in minute doses .... 541

Lawrence's theory of life .......... 447

Lawrence on gonorrhœal rheumatism and ophthalmia .............. 549

Laycock on the iodide of potassium.. 533

Lectures on lithotomy, by Dr. Stevens 219

Lee's history of a female having four mammæ .................... 78

Legal consequences of intellectual mania ...................... 441

Leuret's comparative anatomy of the nervous system ................ 532

Liebig's organic chemistry ......... 524

Life, Hunter's idea of ............ 444

Life, Mr. Lawrence's theory of ...... 447

Life, Tiedemann's theory of ........ 449

Lipoma of large size, successfully extirpated .................... 257

Liquor amnii, chemical examination of 188

Lithotomy, Dr. Stevens' lectures on.. 219

Lithotomy, causes of death from .... 219

Lithotrity, Mr. Fergusson on the dangers of ................. 223

Lithuria ......................... 18

Liver, organic disease of the........ 148

Liver, enlargement of the left lobe of, 199

Liver, atrophy of the .............. 320

Liver, diseases of the .............. 332

Liver, inflammation of the.......... 333

Liver, enlargement or other organic disease of the ................. 333

Liver, hypertrophy of.............. 335

Lizars on surgery ................ 168

Lobar emphysema ................ 399

Lobular emphysema .............. 398

Lombard on pulmonary emphysema, 397

Lower extremities, Mr. Stafford on contraction of the ............. 567

Lunacy, law of ................... 277

Lunatic asylum, at Richmond, report of the ....................... 558

Lunatic asylum, at York, report of the 565

Lunatic asylum at Dundee, report of, 575

Lungs, diseases of the ............. 59

M.

Macartney on inflammation ........ 119
Magendie on the blood ............ 203
Magnesia, pellucid solution of ...... 629
Maitland on the physiology of the blood 69
Malformations of the shoulder and hip-joints ...................... 171
Man, physiology of ............... 443
Mania, bad effects of great reduction in 566
Marshall's, Mr. gleanings in the Indian seas ......................... 633
Mayo's pathology of the human mind, 482
Mechanism versus vitalism ........ 287
Meckel's descriptive and pathological anatomy ..................... 173
Medical society of Bombay, transactions of the..................... 73
Medical annual for 1839 ........... 164
Medical jurisprudence, Mr. Taylor on, 193
Medical profession, studies required for 211
Medical statistics of armies ......... 411
Medical jurisprudence of insanity, by Dr. Ray ...................... 431
Medical portrait gallery ............ 525
Medical and Surgical Society of Newcastle, report of the ............. 648
Medicine, Dr. Thompson on the improvement of .................. 160
Medicine, Sir P. Crampton's history of 626
Medico-chirurgical transactions .... 76
Mental diseases in general .......... 439
Mental disorders, M. Esquirol on.... 37
Mercurial inunction in peritonitis.... 247
Mercury as an antiphlogistic remedy, 245
Mercury in minute doses, Dr. Law on, 541
Military surgery, Sir C. Ballingall on, 408
Milky urine, case of .............. 617
Mollan's report of the Richmond Lunatic Asylum ................... 558
Mondiere on the perforation of the intestines by worms .............. 233
Morbid anatomy of puerperal fever .. 465
Morbus cordis..................... 96
Mortality after amputations ........ 556
Morton's surgical anatomy of the perinæum ....................... 174
Moulding tablets for fractures, Mr. Smee on ....................... 536
Müller's elements of physiology ..... 517
Muscles of the eye, Mr. Cooper on the 201

N.

Natural history ................... 521
Natural medicine ................. 626
Naturalist's Library, by Sir William Jardine ....................... 522
Nature of puerperal fever .......... 466
Necrosis, Mr. Gulliver on .......... 86
Nervous form of puerperal fever .... 464
Nervous system, comparative anatomy of the ........................ 532
Neuralgia, Dr. Rowland on ......... 63
Neuralgia, exciting causes of........ 64
Neuralgia, seat of ................. 65
Neuralgia, pathology of............ 66
Neuralgia, treatment of............ 66
Neuralgia......................... 229
Neuralgia of the larynx............ 539
Neuralgia of the testicle........... 539
Newcastle, report of the Medical and Surgical Society of.............. 649
New weekly contemporary ......... 621

O.

Œdema ......................... 125
Open foramen ovale .............. 194
Opening between the ventricles of the heart ......................... 591

Operation, sudden death after an 259
Oppenheim and Fricke on iodine injections in hydrocele 605
Organic disease of the liver 148
Organic diseases of the stomach 316
Organic chemistry for the chair of, at Paris 594
Organic chemistry, by Professor Liebig 524
Origin of the aorta from the right ventricle 591
Orthopædic establishments in Paris 592
Osiander on puerperal fever 595
Osteology, Mr. Warde on 174
Osteology, Dr. Boisragon's illustrations of 520
Ovarian tumors 200

P.

Pali plague, Dr. Rankin on the 112
Pali plague, description of the 113
Palermo hospital, surgical report of the 258
Palsy 229
Paralysis of the eyelids 613
Parotid gland, extirpation of the 550
Pathology of the human mind, by Dr. Mayo 482
Pathology and treatment of small-pox, Mr. Stephens on the 509
Pauperism and poor-laws, Dr. Fergusson on 504
Pellucid solution of magnesia 626
Pennock's case of anomalous aneurysm of the aorta 545
Pennsylvania hospital, clinical reports from the 554
Perforation of the intestines by worms 233
Perinæum, surgical anatomy of the 174
Peritoneal form of puerperal fever 462
Peritonitis, mercurial inunction in 247
Peritonitis 331
Pettigrew's medical portrait gallery 525
Philosophy of disease, Mr. Harrison on the 510
Phosphatic state of the urine 33
Phthisis 95
Phthisis pulmonalis, caustic issues in 599
Physician, remarkable state of disease in a 647
Physiology of the blood, Dr. Maitland on the 69
Physiology of man 445
Physiology, Muller's elements of 517
Pilcher on the ear 414
Plague, Dr. Bowring on the 163
Pneumo-thorax 232
Pneumo-thorax, Dr. Bigelow on 552
Poisoning by sulphuric acid 79
Poisoning by arsenic 251
Poisoning apparatus of serpents 521
Poisoning by arsenic, successful treatment of 612
Pommade against the falling off of the hair 610
Popliteal aneurysm, Mr. Hadwen's case of 80
Popliteal aneurysm 149
Porter, on puncturing the dura mater after trephining 578
Post mortem examination of a case of dropsy 646
Potassium, Mr. Laycock on iodide of 533
Predisposing causes of gastric affections 305
Pressure to the uterus, Mr. Fenner on 338
Primary treatment of injuries, Dr. Stevens on 216
Prolapsus uteri, Mr. Gray on 346
Prolapsus uteri 610
Pulmonary emphysema, Dr. Lombard 397
Pulmonary emphysema, formations of 399
Pulmonary emphysema, symptoms of 401
Pulmonary emphysema, treatment of 403
Pulmonary consumption, Mr. Hare on 481
Pulmonary diseases, inhalation of iodine in 535
Pulsation in the superficial veins of the arms and neck 237
Pulse, effect on the, by change of posture 182
Puerperal fever, Dr. Fergusson on 461
Puerperal fever, forms of 461
Puerperal fever, peritoneal form of 462
Puerperal fever, nervous form of 464
Puerperal fever, complicated form of 464
Puerperal fever, morbid anatomy of 465
Puerperal fever, nature of 466
Puerperal constitution 470
Puerperal fever, treatment of 472
Puerperal fever, gastro-enteric form of 474
Puerperal fever, treatment of ataxic form of 475
Puerperal fever, M. Osiander on 595
Pus in the blood 273
Pylorus, carcinoma of the 320

Q.

Quackery, Dr. Ticknor on 504

Quain's plates 174

Quarantine laws, Mr. Holroyd on the 525

Quarantines, Mr. Bowring on 163

R

Radius, dislocation of the head of the, backwards 555

Rankin on the Pali plague 112

Ray's medical jurisprudence of insanity 431

Rayer on urinary diseases 1, 350

Rectum, scirrhus of the 431

Rees on animal and earthy matter in human bones 90

Rees' chemical examination of the liquor amnii 188

Rees, on diabetic blood 190

Remarkable state of disease in a physician ........................ 647

Remedial value of carbonate of soda.. 288

Remedies for inflammation ........ 136

Remittent mania ................ 576

Reparation, modes of.............. 128

Reparation, in different tissues ...... 129

Report of the Med. and Surg. Society of Newcastle ................ 649

Reputed consequences of inflammation 126

Richmond lunatic asylum, report of.. 559

Ricord on the use of iron in syphilitic affections..................... 620

Rowland on neuralgia ........... 63

Rupture of the diaphragm.......... 193

S.

Saline alkaline diuretics............ 243
Saratoga springs.................. 281
Scalp, chronic hypertrophy of the .. 170
Scalp, diffuse inflammation of the .. 261
Scholefield, Dr. and Dr. Hooper .... 287
Scirrhous thickening of the stomach 199
Scirrhus of the rectum ............ 331
Scott on Cove ................... 288
Scudamore, Sir C. on gout ........ 530
Seat of neuralgia ................ 65
Secondary syphilis, Dr. Johnston on the salts of silver in ............ 538
Serpents, poisoning apparatus of .... 521
Sharkey on the epidemic cholera of Berehaven ................... 289
Shortening of the neck of the femur, Mr. Gulliver on ............... 543
Shoulder and hip-joints, malformations of the.................... 171
Simulation, curious cases of........ 240
Skin, Mr. Dendy, on diseases of the.. 152
Small-pox ...................... 581
Small-pox, Mr. Stephens on the pathology and treatment of ......... 509
Smee on moulding tablets for fractures 536
Smith's clinical observations on surgery 550
Soda, carbonate of, remedial value of 288
Solid matters of the urine .......... 1
Soupe à la minute ................ 239
Spine, Hare on curvatures of the.... 478
Spine, angular curvature of the .... 481
Spleen, alterations of the substance of the ........................ 194
Spleen, inflammation of the ........ 195
Spleen, purulent deposits in the .... 197
Spleen, malignant disease of the .... 196
Spleen, ulcerations of the tunics of the 197
Stafford on contraction of the lower extremities .................... 567
Stammering, Mr. Bartlett on ...... 507
Stanley's case of anchylosis of cervical vertebræ .................... 296
Stapedius, action of .............. 424
State of the faculty in Algiers ...... 534
State of vaccination .............. 271
Statistical reports from the West Indies 49
Statistics of fever in Glasgow ...... 511
Stephens on the pathology and treatment of small-pox .............. 509
Stevens on the primary treatment of injuries ...................... 216
Stevens's lectures on lithotomy...... 219
St. Lucia........................ 55
Stokes on the use of wine in typhous fever ...................... 579
Stoll on delirium tremens .......... 269
Stomach and bowels, diseases of the.. 60
Stomach, scirrhous thickening of the 199
Stomach and intestines, diseases of the 299
Stomach, derangement of the sensibility of the .................... 300
Stomach, functional derangement of the 299
Stomach, inflammatory and organic diseases of ..................... 316
Stone crushed in bladder 270 years ago 629
Studies required for the medical profession......................... 211
St. Vincents .................... 54
Subclavian artery and vein, irregularities of the .................... 592
Successful extirpation of a lipoma of large size .................... 257
Sudden death after an operation .... 259
Sudden death, M. Devergie on ...... 266
Suicide, connection of insanity with 489
Sulphuric acid, Dr. Wilson on poisoning by ...................... 79
Suppuration, Mr. Gulliver on ...... 273
Surgeon's vade-mecum by Druitt.... 522
Surgery, recent works on .......... 168
Surgery, Mr. Lizars on ............ 169
Surgery, Burn's principles of ...... 169
Surgery, Dr. Smith's clinical observations on ...................... 550
Surgical anatomy of the perinæum .. 174
Surgical report of the Palermo hospital 258
Syme's clinical report............. 572
Symptoms of pulmonary emphysema 401
Syphilitic affections, utility of iron in 620

T.

Taylor on medical jurisprudence .... 193
Temperance societies in America .... 166
Temperance societies .............. 625
Testicle, neuralgia of the .......... 539
Thompson on improvement of medicine 160
Thoracic aorta, aneurysm of the .... 631
Thurnam on aneurysms of the heart.. 102
Ticknor on quackery .............. 404
Tiedeman's theory of life .......... 449
Tobago ........................ 53
Torticollis, case of ................ 253
Toulmouche on chlorine inhalation in catarrh ...................... 231
Transactions of Med. Society of Bombay 73

Traumatic gangrene, successful amputation for........................ 552
Treatment of neuralgia ........... 66
Treatment of incontinence of urine with nux vomica................ 230
Treatment of pulmonary emphysema 403
Treatment of puerperal fever ...... 472
Trinidad ........................ 53
Tumors, encysted ................ 262
Tunics of the spleen, ulcerations of the 197
Turner's elements of chemistry...... 524
Tying polypi of the uterus, new instruments for .................... 167
Typhous fever, Dr. Stokes on the use wine in ...................... 579
Typhous fever, M. Bricheteau on.... 601

U.

Ulcerations of the tunics of the spleen 197
Umbilical vein, inflammation of the, in infants... .................. 247
Urea in the blood ................ 240
Urea in the blood in cholera ..... . 536
Urinary diseases, Dr. Willis on ....1, 350
Urinary diseases, Rayer on ......1, 350
Urine, solid matters of the.......... 4
Urine, phosphatic state of the ...... 33
Urochyalisis senum .............. 15
Uterine hæmorrhage, M. Dubois on.. 611
Uterus, new instruments for tying polype of the .................. 167
Uterus, Mr. Fenner on pressure to the 338
Utility of iron in syphilitic affections 620

V.

Vaccination, state of .............. 271

Vaccination and small-pox ........ 581

Valves of the heart, aneurysm of the 110

Vampirism of the nineteenth century 280

Various diseases, Dr. Graves on .... 539

Ventricles, aneurysm of the ........ 102

Veratria, Dr. Forcke on............ 228

Vesalius, death of ................ 526

Vesical calculus .................. 258

Vesicular emphysema.............. 398

Virtues of the iodide of potassium .. 533

Vital organs in chronic diseases .... 93

Vital principle, discovery of the .... 443

Vital statistics of Glasgow ........ 511

Vitalism versus mechanism ........ 287

W.

Warde's outlines of human osteology 174

Waugh on cerebro-spinal phenomena 174

West Indies, statistical reports from the ......................... 4.

Willis on urinary diseases ........1, 350

Willis' illustrations of cutaneous disease 519

Wilson on poisoning by sulphuric acid 79

Wilson's practical & surgical anatomy 174

Wine in typhous fever ............ 579

Worms, perforation of the intestines by 233

Wry-neck, case of ................ 253

Y.

Yarrel's history of British birds .... 176

Yates on the causes of epidemic fever 162

York lunatic asylum, report of the .. 565

Z.

Zeitschrift fur die gesammte medicin.. 589

---

### TO CORRESPONDENTS.

"Is Cholera contagious?" This communication has been received; but we must decline opening up a question which is now obsolete. In a late meeting of the Royal Medico-Chirurgical Society, where Dr. Budd brought forward the subject on a large statistical scale, not a voice was heard in favour of the contagion of cholera! Why then agitate the question more? Even if the doctrine were proved, its withering influence in case of another outbreak would be productive of dreadful evil—and if the doctrine be untenable, which we believe it is—how unjustifiable to scatter doubts on the subject. We do not like to incur the responsibility. The paper will be delivered to the author, or any other person whom he may depute to receive it.

Dr. Churchill's paper will be attended to in our next.

Mr. Fiddes' communication was received too late for the present number.

To Palazzo and several other querists we have to state that we know nothing of the Court transaction alluded to, beyond the rumours which have been publicly disseminated in the newspapers.

